# Characterisation of the broad substrate specificity 2-keto acid decarboxylase Aro10p of *Saccharomyces kudriavzevii* and its implication in aroma development

**DOI:** 10.1186/s12934-016-0449-z

**Published:** 2016-03-12

**Authors:** Jiri Stribny, Gabriele Romagnoli, Roberto Pérez-Torrado, Jean-Marc Daran, Amparo Querol

**Affiliations:** Food Biotechnology Department, Institute of Agrochemistry and Food Technology, (IATA-CSIC) Avda, Agustín Escardino, 7, Paterna, 46980 Valencia, Spain; Department of Biotechnology, Delft University of Technology, Delft, The Netherlands; Kluyver Centre for Genomics of Industrial Fermentation, Delft, The Netherlands; Platform Green Synthetic Biology, Delft, The Netherlands

**Keywords:** *Saccharomyces kudriavzevii*, *S. cerevisiae*, *ARO10*, 2-keto acid decarboxylase, Amino acid catabolism, Higher alcohols, Acetate esters, Grantham matrix

## Abstract

**Background:**

The yeast amino acid catabolism plays an important role in flavour generation since higher alcohols and acetate esters, amino acid catabolism end products, are key components of overall flavour and aroma in fermented products. Comparative studies have shown that other *Saccharomyces* species, such as *S. kudriavzevii*, differ during the production of aroma-active higher alcohols and their esters compared to *S. cerevisiae*.

**Results:**

In this study, we performed a comparative analysis of the enzymes involved in the amino acid catabolism of *S. kudriavzevii* with their potential to improve the flavour production capacity of *S. cerevisiae*. In silico screening, based on the severity of amino acid substitutions evaluated by Grantham matrix, revealed four candidates, of which *S. kudriavzevii* Aro10p (SkAro10p) had the highest score. The analysis of higher alcohols and esters produced by *S. cerevisiae* then revealed enhanced formation of isobutanol, isoamyl alcohol and their esters when endogenous *ARO10* was replaced with *ARO10* from *S. kudriavzevii.* Also, significant differences in the aroma profile were found in fermentations of synthetic wine must. Substrate specificities of SkAro10p were compared with those of *S. cerevisiae* Aro10p (ScAro10p) by their expression in a 2-keto acid decarboxylase-null *S. cerevisiae* strain. Unlike the cell extracts with expressed ScAro10p which showed greater activity for phenylpyruvate, which suggests this phenylalanine-derivative to be the preferred substrate, the decarboxylation activities measured in the cell extracts with SkAro10p ranged with all the tested substrates at the same level. The activities of SkAro10p towards substrates (except phenylpyruvate) were higher than of those for ScAro10p.

**Conclusions:**

The results indicate that the amino acid variations observed between the orthologues decarboxylases encoded by *SkARO10* and *ScARO10* could be the reason for the distinct enzyme properties, which possibly lead to the enhanced production of several flavour compounds. The knowledge on the important enzyme involved in higher alcohols biosynthesis by *S. kudriavzevii* could be of scientific as well as of applied interest.

**Electronic supplementary material:**

The online version of this article (doi:10.1186/s12934-016-0449-z) contains supplementary material, which is available to authorized users.

## Background

Higher alcohols and acetate esters (compounds naturally produced by yeast metabolism during fermentation) belong to the most important contributors to the organoleptic properties of a wide range of fermented beverages and foods, and are important components in the cosmetic industry [1]. The most significant acetate esters, i.e. isobutyl acetate (fruity-like aroma), isoamyl acetate (banana), and 2-phenylethyl acetate (flowery, rose-like), are products of a condensation reaction between the corresponding higher alcohol (isobutanol, isoamyl alcohol, 2-phenylethanol, respectively) and acetyl-CoA [2, 3]. In *Saccharomyces cerevisiae*, the predominant yeast in food-related fermentations, depending on the conditions these higher alcohols are synthesised from 2-keto acids derived either from glycolysis or from the catabolism of valine, leucine and phenylalanine [4–7] on a reaction pathway also known as the Ehrlich pathway [8, 9]. On this pathway, the amino acids, which are transported by amino acid permeases (codified by *GAP1, BAP2, BAP3, MUP3*) [10–13] are first transaminated to the corresponding 2-keto acids by transaminases (codified by *BAT1, BAT2, ARO8, ARO9*) [14–16]. These 2-keto acids are then decarboxylated by decarboxylases (codified by *PDC1, PDC5, PDC6, ARO10*) [17, 18]. The resulting aldehydes are reduced to their corresponding alcohols by dehydrogenases (codified by *ADH1*-*7, SFA1*) [4]. The subsequent acetate ester formation is mediated by the alcohol acetyltransferases codified by genes *ATF1* and *ATF2* [19, 20]. Conversely, acetate ester breakdown is affected by the function of hydrolases, such as those encoded by Iah1p [21] which, together with Atf1p and Atf2p, maintain an optimal ester accumulation rate.

Apart from the commonly used *S. cerevisiae*, other yeasts are being investigated as being potential to tailor and improve food-related processes, such as winemaking, including flavour substances production. We recently reported differences in the production of prime aroma-active compounds between *S. cerevisiae* and *S.kudriavzevii* [22]. Since *S. kudriavzevii* is characterized as a cryotolerant species, improved flavour compounds production is usually explained by low-temperature fermentation [23–25]. Nevertheless our work [22], in which several nitrogen sources were used, including individual amino acids valine, leucine, and phenylalanine as the precursors of higher alcohols and acetate esters, revealed that, for instance, *S.kudriavzevii* produced larger amounts of higher alcohols than *S. cerevisiae,* even at 25 °C.

To better understand the aforementioned differences, the present study aimed to explore nucleotide divergences in the genes (and consequently in the corresponding enzymes) involved in flavour compounds production. To achieve this we used the Grantham scoring, which quantitatively evaluates (dis) similarity in amino acids substitutions on the basis of physiochemical properties (composition, polarity and molecular volume), and according to increasing biochemical dissimilarity classifies the amino acids substitutions as conservative or radical [26, 27]. By using this tool, we searched for the *S. kudriavzevii* genes that encode enzymes whose amino acid sequences have the most radical changes compared to *S. cerevisiae*. The bioinformatic analysis revealed *ARO10*, which codifies a broad-substrate-specificity 2-keto acid decarboxylase [28], to be the candidate with the highest score for radical changes. Thus we cloned *S. kudriavzevii ARO10* (*SkARO10*) into *S. cerevisiae* to examine its impact on the production of higher alcohols and acetate esters. The substrate specificities and kinetic properties of the encoded enzyme were also analysed and compared to *S. cerevisiae*.

## Results

### In silico analysis revealed the largest amount of radical amino acid substitutions between the Aro10p orthologues

To perform a comparative analysis, DNA sequences of the orthologue genes encoding 23 enzymes which are involved in amino acid catabolism leading to higher alcohols and acetate ester formation were obtained from 75 *S. cerevisiae* strains and two *S. kudriavzevii* strains, all available in databases (Additional File 1). Amino acid translations of the DNA sequences were then aligned. These alignments allowed us to search for amino acid substitutions between orthologues. The individual changes in *S. kudriavzevii* sequences (with *S. cerevisiae* orthologues taken as references) were then quantified by Grantham matrix, which scores the difference between two amino acids according to composition, polarity and molecular volume. Substitutions with a score of 120 and higher were considered radical. Across the 23 assessed sequences, three were evaluated with significantly higher Grantham scores for the total substitutions: 2-keto acid decarboxylase encoded by *ARO10*, and two alcohol acetyltransferases encoded by *ATF1* and *ATF2* (Table 1). These three sequences, with total Grantham scores of 5764, 5350 and 6187, respectively, surpassed the other two highest sequences (Aro9p–3560, Bap2p–3350) by about 40 %. The highest total Grantham score and the largest amount of substitutions (110) were found in Atf2p. However, the largest amount of radical substitutions contained Aro10p (11 substitutions) with a score of 1629, while Atf1p and Atf2p contained six and four with a score of 942 and 609, respectively. The combination of the highest Grantham score for the radical substitutions and the second highest score for all the substitutions left *ARO10* as the candidate selected for further assays.

**Table 1:** Amino acid substitutions in the orthologous enzymes from *S. kudriavzevii* and *S. cerevisiae* evaluated by the Grantham score

Name	AAs	Total substitutions		Radical substitutions (≥120)		$\frac{{\sum {\text{Gr}\text{.}\,\text{score}\,\text{of}\,\text{radicals}} }}{{\sum {\text{Gr}\text{.}\,\text{score}\,\text{total}} }}* 100\,{(\% )}$
		No.	∑ Grantham score	No.	∑ Grantham score	
*Permeases*						
Gap1	602	34	2050	2	275	13.4
Bap2	609	58	3350	6	797	23.8
Bap3	604	45	2276	2	301	13.2
Mup3	546	49	2500	3	478	19.1
*Transaminases*						
Bat1	393	18	817	0	0	0
Bat2	376	41	2640	4	649	24.6
Aro8	500	34	2154	1	180	8.4
Aro9	517	72	3560	3	421	11.8
*Decarboxylases*						
Pdc1	563	10	567	1	125	22.0
Pdc5	563	30	1266	0	0	0
Pdc6	563	NF	–	–	–	–
Aro10	635	87	5764	11	1629	28.3
*Dehydrogenases*						
Adh1	348	15	727	0	0	0
Adh2	348	22	812	0	0	0
Adh3	375	15	821	1	149	18.1
Adh4	382	31	1602	0	0	0
Adh5	351	25	1172	0	0	0
Adh6	360	29	1428	0	0	0
Adh7	361	40	2605	4	584	22.4
Sfa1	385	28	1640	5	700	42.7
*Acetyltransferases*						
Atf1	524	89	5350	6	942	17.6
Atf2	535	110	6187	4	609	9.8
*Esterase*						
Iah1	238	49	2849	3	449	15.8

The corresponding enzymes are involved in the production of aroma-active higher alcohols and acetate esters

NF sequence not found in the *S. kudriavzevii* database

Radical substitutions involve each substitution with a Grantham score ≥120

It is worth mentioning that, to date, there are publicly available genome sequences from two *S. kudriavzevii* strains (IFO1802 and ZP591) [29]. Since the analysis revealed only minor differences between these two strains (e.g. no differences were observed in the selected *ARO10*), type strain IFO1802 was used for further experiments.

### Effect of *SkARO10* on the formation of the higher alcohols and esters

**Fig. 1:**
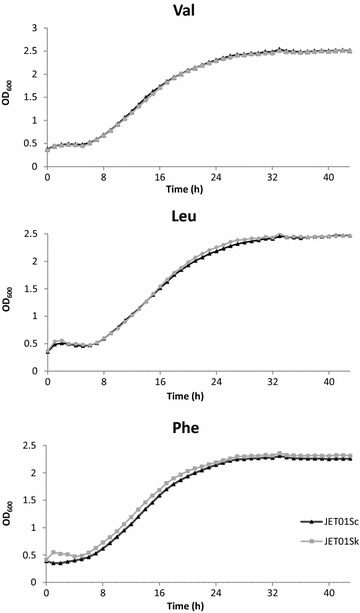
Growth of JET01Sk and JET01Sc with the indicated amino acids as the nitrogen source

**Fig. 2:**
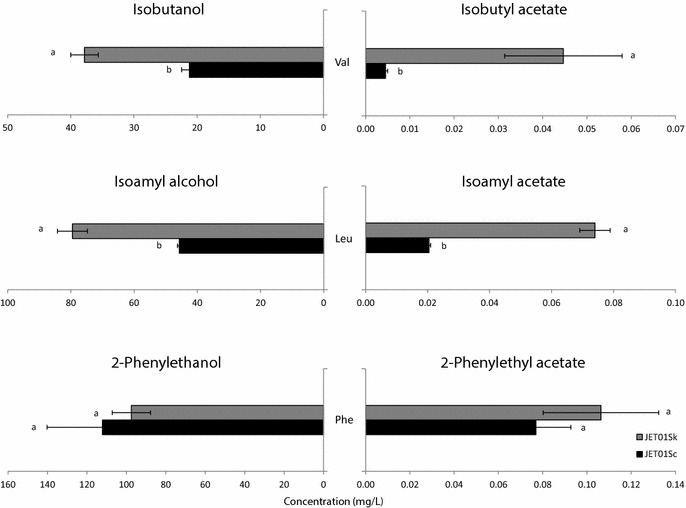
Production of the higher alcohols and esters derived from the corresponding amino acidic precursors which were used as the nitrogen source. The corresponding amino acids are indicated between the *bars*. The statistically significant differences among the species were determined independently for each nitrogen source and are indicated by labels beside the *columns*

In order to verify the impact of *S. kudriavzeviiARO10* (*SkARO10*) on the production of higher alcohols and/or acetate esters, the native *ARO10* (*ScARO10*) allele of a haploid strain of the wine *S. cerevisiae* T73 strain was swapped with *SkARO10* allele resulting in the mutant strain JET01Sk (Table 2). To exclude any other mutations that may have occurred during the allele replacement step, the original *ScARO10* allele was introduced back at its native position resulting in the strain JET01Sc. Subsequently, the formation of the major aroma-active higher alcohols and acetate esters was measured and compared between JET01Sc and JET01Sk. Cells were cultivated with individual amino acids valine, leucine or phenylalanine as the sole nitrogen source, and the corresponding aroma-active higher alcohols and their esters were analysed. Such medium with defined amino acids as the sole nitrogen source allowed us to observe the in vivo effect of the *SkARO10* allele on the production of valine-, leucine-, and phenylalanine-derived higher alcohols and their esters with no undesirable impact of other non-specific nitrogen sources. Under the tested conditions, both strains exhibited a normal growth with no significant differences among them (Fig. 1). This confirmed that both decarboxylases showed activity with the formed 2-keto acids, enabling the strains to use these amino acids as the sole nitrogen source. With valine as the nitrogen source, both corresponding derivatives (isobutanol and isobutyl acetate) were produced by the strain that carried the *SkARO10* allele in significantly larger amounts (Fig. 2). The isobutyl acetate concentration was *c.* 9-fold higher than that produced by JET01Sc. A similar upward trend in favour of JET01Sk was observed in the production of isoamyl alcohol and isoamyl acetate when their amino acidic precursor leucine was used as the nitrogen source. JET01Sk showed an almost 2-fold and 3.6-fold increase in the isoamyl alcohol and the isoamyl acetate concentration, respectively. The phenylalanine-grown cultures exhibited slight, but statistically insignificant, differences during the formation of the corresponding products, these being 2-phenylethanol and 2-phenylethyl acetate.

**Table 2:** List of the yeast strains used in this study

Strain	Species	Description	Reference
T73	*S. cerevisiae*	Wine strain, Alicante, Spain	[49]
IFO1802	*S. kudriavzevii*	Type strain, NCBI	[50]
Ta	*S. cerevisiae*	T73*ho∆::loxP*	A. Querol
JET01	*S. cerevisiae*	Ta *aro10*∆::*NAT1*	This study
JET01Sk	*S. cerevisiae*	Ta *aro10*∆::*SkARO10*-*kX*	This study
JET01Sc	*S. cerevisiae*	Ta aro10∆::*ScARO10*-*kX*	This study
CEN.PK 711-7C	*S. cerevisiae*	*MAT*a *ura3*-*52* *pdc1*∆ *pdc5*∆ *pdc6*∆ *aro10*∆ *thi3*∆	[28]
CEN.PKpSkARO10	*S. cerevisiae*	CEN.PK 711-7C pG-SkARO10-kX	This study
CEN.PKpScARO10	*S. cerevisiae*	CEN.PK 711-7C pG-ScARO10-kX	This study

**Fig. 3:**
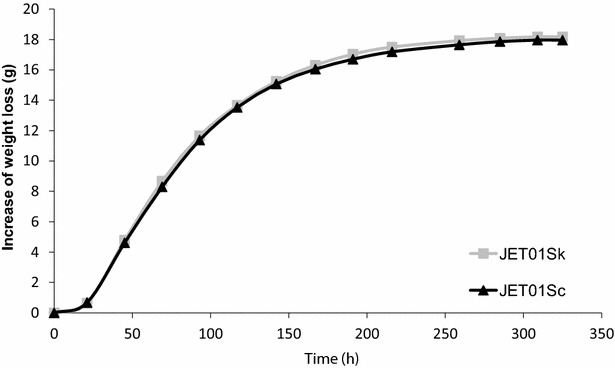
Progress of synthetic wine must fermentation. The fermentations were monitored by weight loss until the constant weight was achieved

**Fig. 4:**
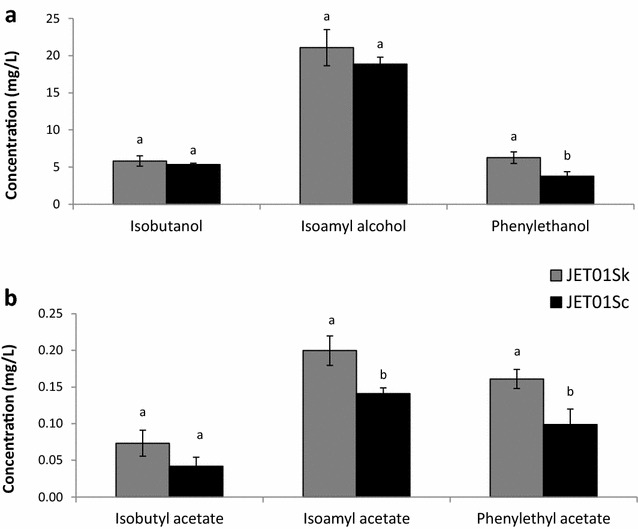
Production of the higher alcohols (**a**) and acetate esters (**b**) by JET01Sk and JET01Sc during the fermentation of the synthetic wine must. The statistically significant differences among the species were determined independently for each nitrogen source and are indicated by *labels* above the *columns*

Additionally, in order to verify the effect of the *SkARO10* allele on the formation of higher alcohols and esters in a more complex medium, fermentations of the synthetic wine must by the strains JET01Sk and JET01Sc were performed, and the production of higher alcohols and acetate esters was analysed. Weight loss monitoring revealed that both strains exhibited similar fermentations rates with no differences (Fig. 3). Interestingly, regarding the higher alcohols, JET01Sk only exhibited an increased amount of 2-phenylethanol (Fig. 4a) which is in contrast with the data observed in the cultivations with the individual amino acids as the nitrogen source. Regarding the acetate esters, JET01Sk showed larger amounts of isoamyl acetate and 2-phenylethyl acetate when compared to JET01Sc (Fig. 4b).

### Comparison of the substrate specificity of SkAro10p and ScAro10p

**Fig. 5:**
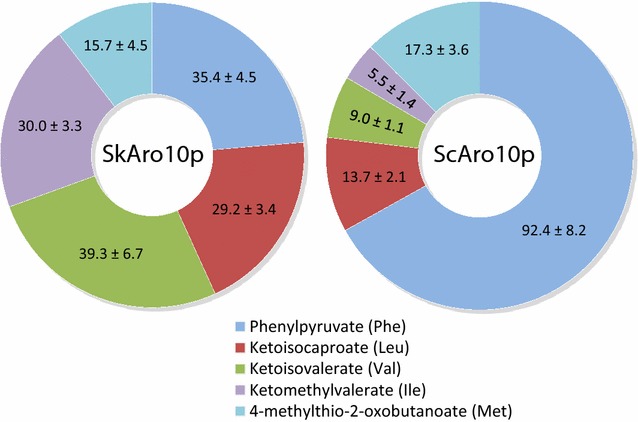
Activities [nmol min^−1^∙(mg protein)^−1^] of SkAro10p and ScAro10p measured in the cell extracts of *S. cerevisiae* strain with *pdc1 pdc5 pdc6 aro10 thi3* deletion. Activities were measured at a concentration of 5 mM for phenylpyruvate and at 10 mM for the other substrates. The amino acidic precursors of the corresponding substrates are offered in the parentheses following the substrates

To relate these metabolites concentration differences to the presence of either *SkRO10* or *ScARO10* the substrate specificity of SkAro10p and ScAro10p were compared. To achieve this, the individual decarboxylase genes were expressed in a host *S. cerevisiae* strain that lacked all the 2-keto acid decarboxylase genes involved in the decarboxylation step of the Ehrlich pathway (CEN.PK711-7C *pdc1∆ pdc5∆ pdc6∆ aro10∆ thi3∆*). Absence of the pyruvate decarboxylase genes (*PDC1, PDC5, PDC6*) has been previously shown to inhibit growth on glucose [30]. Therefore, ethanol was used as a carbon source in the chemostat cultivations. Eventually, to overcome the tight transcriptional control of *ARO10* gene [31] and the regulation of the Aro10p activity by the nitrogen sources [28], phenylalanine was used as the nitrogen source rather than ammonium sulphate. Decarboxylase activity was measured in the cell extracts from the chemostat cultures and was compared for five different substrates phenylpyruvate, ketoisocaproate, ketoisovalerate, ketomethylvalerate, and 4-methylthio-2-oxobutanoate. Substrates were used at saturating concentrations of 10 mM, except for phenylpyruvate (5 mM). The cell extracts of both strains (CEN.PKpSkARO10 and CEN.PKpScARO10) exhibited activities for all five substrates (Fig. 5). Nevertheless, when comparing the individual substrates, the strain that carried *ScARO10* displayed significantly greater activity for phenylpyruvate than for the other substrates. In contrast, the cell extracts of the strain that expressed *SkARO10* exhibited similar activities for all substrates.

Furthermore, the kinetic properties of the two decarboxylases were assessed for phenylpyruvate. The typical Michaelis–Menten saturation kinetics was observed for the enzymatic activities measured in the cell extracts of both strains. The *K*_*m*_ for this substrate was 2-fold lower for *S. kudriavzevii* Aro10p than the *K*_*m*_ found for *S. cerevisiae* Aro10p (0.07 ± 0.005 vs. 0.15 ± 0.01 mM, respectively) and SkAro10p showed 3-fold lower *V*_*max*_ than ScAro10p [6.95 ± 0.15 vs. 21.7 ± 0.5 nmol min^−1^ (mg protein)^−1^].

## Discussion

In previous works, significant differences were found between *S. kudriavzevii* and *S. cerevisiae* in the production of aroma-active compounds during the fermentations of natural media (particularly wine must) or in synthetic media [23, 32–34]. Particularly, *S. kudriavzevii* surpassed *S. cerevisiae* for aroma-active higher alcohols production. However, *S. cerevisiae* is the main species used in many industrial processes. The knowledge of the genetic aspects of the aroma production by *S. kudriavzevii* could prove useful for tailoring already used *S. cerevisiae* strains that could lead to aroma production improvement.

We applied here Grantham scoring, based on the assessment of biochemical dissimilarity between amino acid side chain properties [26, 27], to identify the most radical non-synonymous nucleotide changes in the orthologues genes from *S. kudriavzevii* vs. *S. cerevisiae* that encode the enzymes from the amino acid catabolism pathway leading to aroma-active higher alcohols and the corresponding acetate esters. The largest number of radical substitutions was observed in *SkARO10* (Table 1). The analysis revealed 11 of the 87 substitutions as being radical, which is *c.* 12.5 %. When expressing these substitutions as a Grantham score, the ratio was 1629 of 5764, thus *c.* 28 %. However, the highest ratio of radical vs. total substitution was observed in Sfa1p, which is a bifunctional enzyme that displays the glutathione-dependent formaldehyde dehydrogenase activity required for formaldehyde detoxification, and the alcohol dehydrogenase activity involved in the formation of higher alcohols [35]. In spite of the fact that the radical substitutions represented only 5 of 28, according to the Grantham scale, these five substitutions comprise *c.* 43 % of the Grantham score for all the substitutions. This suggests that Sfa1p is a good result. Nevertheless, in this study we first focused on *SkARO10* as the result with the highest Grantham score for radical changes. The impact of *SkSFA1*, *SkATF1* and *SkATF2* will be investigated in further studies.

In *S. cerevisiae*, the product of the *ARO10* gene was described as a 2-keto acid decarboxylase which catalyses the decarboxylation of the 2-keto acids derived from the amino acid transamination on the Ehrlich pathway [18]. This decarboxylation step is the only irreversible reaction that takes place on the Ehrlich pathway. Besides Aro10p, other decarboxylases (Pdc1p, Pdc5p, Pdc6p) also catalyse the conversion of 2-keto acids into the corresponding aldehydes. Nevertheless, Aro10p showed superior kinetic parameters for branched-chain, aromatic, and sulphur-containing 2-keto acids than the other decarboxylases [36]. This broad-substrate specificity, together with the kinetic properties suggested that Aro10p was the major decarboxylase involved in the formation of the aroma-active higher alcohols that derived from the branched-chain, aromatic and sulphur-containing aroma acids [36].

The enzymatic activities of SkAro10p, assayed in a *pdc1 pdc5 pdc6 aro10 thi3* quintuple-null *S. cerevisiae* strain, were observed for all the tested substrates. This result indicates that the substrate specificity of SkAro10p is as broad as that of ScAro10p. Yet significant differences between SkAro10p and ScAro10p were observed for substrate preferences. When individual enzymatic activities were expressed as a percentage distributed among the total enzymatic activity of the measured substrates, SkAro10p was found to be more or less evenly proportional of the enzymatic activities. In contrast, ScAro10p showed considerably greater activity towards phenylpyruvate than the other substrates. This suggests phenylpyruvate to be a preferred substrate. This phenylpyruvate-preference of Aro10p from the wine *S. cerevisiae* T73 strain was consistent with previously observed data for Aro10p from the laboratory *S. cerevisiae* CEN.PK113-7D strain [36], and for the Aro10 isoenzymes encoded by the two (*S. cerevisiae*-derived and *S. eubayanus*-derived) subgenomes of the lager-brewing *S. pastorianus* strain [37].

The impact of *SkARO10* on the production of higher alcohols and their esters was analysed by heterologous expression in a host *S.cerevisiae*. The results showed a remarkable increase in the detected amounts of valine- and leucine-derived higher alcohols (isobutanol and isoamyl alcohol, respectively) produced by the strain that carried *SkARO10* compared to the isogenic reference strain with active *ScARO10*. However, no differences were observed in the formation of 2-phenylethanol from phenylalanine. This phenomenon might be explained by the aforementioned differences in substrate specificities, particularly the phenylpyruvate-preference of ScAro10p. With its strong activity towards phenylpyruvate, ScAro10p produced more 2-phenylethanol and fewer other higher alcohols. Apparently SkAro10p also was sufficiently effective with ketoisovalerate and ketoisocaproate and, therefore, the production of isobutanol and isoamyl alcohol, respectively, was greater than in ScAro10p. The fermentations of synthetic must revealed an opposite result with the differences in 2-phenylethanol formation and no significant differences in the production of isobutanol and isoamyl alcohol. This discrepancy might be due to the complexity of the synthetic must and a possible impact of other compounds on the formation of the higher alcohols.

Similarly, the *S. cerevisiae* that harboured *SkARO10* yielded larger amounts of acetate esters. This improved acetate ester formation was probably the result of the larger production of higher alcohols as they are precursors. Yet in our previous study [22], despite the fact that *S. kudriavzevii* produced larger amounts of higher alcohols than *S. cerevisiae*, *S. kudriavzevii* did not produce larger amounts of acetate esters. This indicates interspecific variations in acetate ester formation; for instance, it has been previously described in *S. cerevisiae* that two alcohol acetate transferases (Atf1p and Atf2p), the enzymes that catalyse the esterification of higher alcohols by acetyl coenzyme A, act differently during ester production. It has been shown that Atf2p plays a minor role in ester formation compared to Atf1p [38]. In *S. kudriavzevii*, the roles of Atf1p and Atf2p, and their substrate specificities, might differ from *S. cerevisiae*. Hypothetically speaking, one possible explanation might be the amino acid variations in the orthologous Atf1p and Atf2p between *S. cerevisiae* and *S. kudriavzevii* observed in our sequence analysis. The suggested hypotheses, together with the provided in silico sequence comparison, indicate that both *ATF1* and *ATF2*, as well as *SFA1* from *S. kudriavzevii,* are good targets for future studies, which would clarify their potential to enhance biotechnological flavour production.

## Conclusions

In this study we detected 2-keto acid decarboxylase (Aro10p) from *S. kudriavzevii* as the possible aspirant to modify the aroma production capacity of *S. cerevisiae*. The heterologous *SkARO10* expression in a host *S. cerevisiae* resulted in increased production of isobutanol and isoamyl alcohol, and their acetate esters, when their amino acidic precursors were used as the nitrogen source. Significant differences in the aroma profile were also found during the fermentations of synthetic must. The analysis of decarboxylase activities in cell extracts revealed remarkable differences between SkAro10p and ScAro10p. Although both enzymes indicated similarly broad substrate specificity, ScAro10p showed a marked preference for phenylpyruvate (the precursor of 2-phenylethanol that confers a rose-like flavour), while the activities of SkAro10p for all the tested substrates were more or less equal. Hence employment of SkAro10p could lead to an overall aroma with a new flavour composition and a more complex profile.

## Methods

### Yeast strains

The yeast strains used in this study are listed in Table 2. *S. cerevisiae* Ta, a haploid strain that derives from commercial wine strain T73, was previously constructed in the laboratory of A. Querol. Stock cultures were grown on standard complex media (0.5 % peptone, 2 % glucose, 0.5 % yeast extract) or on SC-Ura medium [6.7 % YNB, 2 % glucose, 1.92 g/L Drop-out-Ura (Formedium, Norfolk, UK)]. Standard genetic techniques were followed for plasmid and chromosomal DNA isolation, restriction and gel electrophoresis. Strains were transformed by the lithium acetate procedure [39].

### Bioinformatic analysis

To obtain DNA and protein sequences from various *S. cerevisiae* strains and *S. kudriavzevii* strains, several publicly available databases were used: SGD—*Saccharomyces* Genome Database (http://www.yeastgenome.org) [40], SGRP—*Saccharomyces* Genome Resequencing Project (http://www.moseslab.csb.utoronto.ca/sgrp) [41, 42], NCBI (http://www.ncbi.nlm.nih.gov), *Saccharomyces* sensu stricto database (http://www.saccharomycessensustricto.org) [29]. The strains used in the analysis are listed in Additional File 1. Multiple sequence alignments were performed with MEGA 5.05 using the MUSCLE software [43]. The quantification of the amino acid substitutions between the *S. cerevisiae* and *S. kudriavzevii* protein sequences was performed using the Grantham score [26].

### Plasmid and strain construction

The *S.kudriavzeviiARO10* allele (*SkARO10*) was amplified from the genomic DNA of *S.kudriavzevii* IFO1802 using primers SkARO10-aF and SkARO10-aR (Table 3). Primers were designed based on the publicly available sequence of *S. kudriavzevii* IFO1802. The PCR fragment was cloned into the pGREG526 vector [44], previously cut with *Not*I/*Sal*I, and resulted in plasmid pG-SkARO10-kX. The *S.cerevisiaeARO10* allele (*ScARO10*) was PCR-amplified from the genomic DNA of *S.cerevisiae* T73 using primers ScARO10-F/ScARO10-R. The resulting fragment was cloned in NotI-/SalI-digested pGREG526. The plasmid was named pG-ScARO10-kX. The constructed plasmids were then introduced into the CEN.PK711-7C strain, which resulted in strains CEN.PKpSkARO10 and CEN.PKpScARO10.

**Table 3:** Primers used in this study

Primer	Sequence 5′–3′
Cloning into pGREG526	
SkARO10-aF	CCTAGTACGGATTAGAAGCCGCCGAGCGGGTGACAACTTTTGATTTGTTCCCCGC
SkARO10-aR	GCGTGACATAACTAATTACATGACTCGAGGTCGACAAAGACAAAATCGGCGGC
ScARO10-F	CCTAGTACGGATTAGAAGCCGCCGAGCGGGTGACAATCTCTTAGGCATGCTCTTGG
ScARO10-R	GCGTGACATAACTAATTACATGACTCGAGGTCGACTATAATTGCGCCCACAAGTTTC
*ARO10* deletion cassette	
TaARO10-NAT1-F	ATGGCACCTGTTACAATTGAAAAGTTCGTAAATCAAGAAGGGTGTTTAGGTCGATGCCATC
TaARO10-NAT1-R	CTATTTTTTATTTCTTTTAAGTGCCGCTGCTTCAACCATGGGATGGCGGCGTTAGTATCG
Integration fragment	
pGSkARO10f	TAAAGTTTATTTACAAGATAACAAAGAAACTCCCTTAAGCATGACGCCTGTTACAATTAA
pGScARO10f	TAAAGTTTATTTACAAGATAAC
pG-ARO10-R	ACAATTGGTAGCAGTGTTTTATAATTGCGCCCACAAGTTTCTCACTATAGGGCGAATTGG
Diagnostic	
T73AR10-UF	ATCTCTTAGGCATGCTCTTGG
K2	GGGACAATTCAACGCGTCTG
K3	CCTCGACATCATCTGCCC
ScARO10-R1	GAAGTCACCAGGAACACCG
SkARO10-R1	CATTGGAAACAAGGTGCGG

The strategy of replacing endogenous *ARO10* with *SkARO10* in the Ta genome involved two steps i) deletion of the *ARO10* gene and ii) integration of *SkARO10* into the locus.

The *ARO10* gene deletion in the Ta genome was performed by integrating a nourseothricin resistance cassette by homologous recombination. The deletion cassette was amplified using pAG25 [45] as a template and specific primers (Table 3). The resulting strain was named JET01. The integration cassette was amplified from plasmid pG-SkARO10-kX with primers pGSkARO10f and pG-ARO10-R. The resulting PCR fragment included the SkARO10 allele, followed by a kanamycin resistance marker, which was used in the subsequent transformation of the JET01 strain. The final Ta mutant that held the *SkARO10* allele was named JET01Sk. The same procedure was performed with *ScARO10,* which resulted in the restoration of the endogenous allele by the undergone process. This strain, named JET01Sc, was used as a reference in the assays.

### Cultivation to study the production of higher alcohols and acetate esters that derived from the corresponding amino acids

Cultivations were performed in triplicate using a synthetic medium that contained 0.17 % YNB w/o AAs and (NH_4_)_2_SO_4_ (BD DIFCO™, Madrid, Spain) and 2 % glucose as the carbon source, as previously described [22], but with minimal modifications. Media were supplemented by individual amino acids leucine, phenylalanine and valine as the nitrogen source. Concentrations were proportional to 5 g/L (NH_4_)_2_SO_4_ to obtain the same nitrogen content as follows: 10 g/L leucine, 12.5 g/L phenylalanine, 8.9 g/L valine [37].

Starter cultures were prepared by pregrowing yeast in 15-mL tubes that contained 4 mL of standard complex media. Before inoculating the experimental culture, the grown precultures were washed with water and resuspended in the same synthetic medium (with a certain nitrogen source), as used in the assay. Cells were resuspended in such a volume to achieve an OD_600_ of 1.7. These precultures (100 μL) were used to inoculate 1.6 mL of the synthetic media. At this stage the initial OD_600_ was 0.1. Cultivation was performed in 96-well plates with 2-mL-deep wells. Wells were covered by a transparent microplate sealer (Greiner bio-one, Frickenhausen, Germany) to avoid evaporation and loss of volatile flavour compounds. Cultures were incubated for 5 days at 25 °C. The individual 1.7-mL cultures were later transferred to 2-mL tubes and stored at −20 °C for the analysis.

### Yeast growth analysis

Yeast cell growth was followed using a 96-well plate. Synthetic media were supplemented with the amino acids as described above. Then 100 μl of media were inoculated in a well with 2 μl of cell suspension with OD_600_ = 1. Growth was monitored in a Spectrostar Nano absorbance reader (BMG Labtech, Ortenbert, Germany).

### Synthetic wine must fermentation

A synthetic wine must was prepared according to [46], but with 200 g/L of reducing sugars (100 g/L glucose + 100 g/L fructose) and without anaerobic factors [47]. Total nitrogen source 300 mg N/L was a mixture of NH_4_Cl (120 mg/L) and amino acids (180 mg/L). The composition of the amino acids mixture was as described by [47]. The following mineral salts were used: KH_2_PO_4_ 750 mg/L, K_2_SO_4_ 500 mg/L, MgSO_4_ 250 mg/L, CaCl_2_ 155 mg/L, NaCl 200 mg/L, MnSO_4_ 4 mg/L, ZnSO_4_ 4 mg/L, CuSO_4_ 1 mg/L, KI 1 mg/L, CoCl_2_ 0.4 mg/L, H_3_BO_3_ 1 mg/L, (NH_4_)_6_Mo_7_O_24_ 1 mg/L. The following organic acids were used: malic acid 5 g/L, citric acid 0.5 g/L, and tartaric acid 3 g/L. The following vitamins were used: myo-inositol 20 mg/L, calcium panthothenate 1.5 mg/L, nicotinic acid 2 mg/L, chlorohydrate thiamine 0.25 mg/L, chlorohydrate pyridoxine 0.25 mg/L, and biotine 0.003 mg/L. The final pH was adjusted to 3.3 with NaOH.

Fermentations were performed in 250-mL glass bottles containing 200 mL of synthetic must. Fermentations were done in triplicate at 25 °C with continuous orbital shaking (150 rpm). Flasks were closed with Müller valves and monitored by weight loss until a constant weight was obtained. Immediately after the end of fermentation, yeast cells were removed by centrifugation and the content of higher alcohols and esters in the supernatants were analysed by gas chromatography.

### Determination of higher alcohols and esters

The samples stored in the 2-mL tubes were centrifuged (13,000 rpm, 2 min) and 1.5 mL of the supernatant was transferred to 15-mL vials with 0.35 g of NaCl. The 20-μl volume of 2-heptanone (0.005 %) was added as an internal standard. Higher alcohols and esters were analysed by the headspace solid phase microextraction (HS-SPME) technique with a 100-μm poly-dimethylsiloxane (PDMS) fibre (Supelco, Sigma-Aldrich, Madrid, Spain). Solutions were maintained for 2 h at 25 °C to establish the headspace-liquid equilibrium. The fibre was inserted into the headspace through a vial septum and was held for 7 min. The fibre was then inserted into the gas chromatograph inlet port for 4 min at 220 °C with helium flow (1 mL/min) to desorb analytes. A Thermo Science TRACE GC Ultra gas chromatograph with a flame ionization detector (FID) was used, equipped with an HP INNOWax 30 m × 0.25 m capillary column coated with a 0.25-m layer of cross-linked polyethylene glycol (Agilent Technologies, Valencia, Spain). The oven temperature programme was: 5 min at 35 °C, 2 °C/min to 150 °C, 20 °C/min to 250 °C and 2 min at 250 °C. The detector temperature was kept constant at 300 °C. A chromatographic signal was recorded by the ChromQuest programme. Volatile compounds were identified by the retention time for reference compounds. Quantification of the volatile compounds was determined using the calibration graphs of the corresponding standard volatile compounds.

### Enzyme activity measurements

Enzyme activities were assayed in the cell extracts prepared from aerobic ethanol-limited chemostat cultures. Phenylpyruvate, ketoisocaproate, ketoisovalerate, ketomethylvalerate or 4-methylthio-2-oxobutanoate were used as substrates for the enzyme reaction. In order to measure and compare enzyme activity for the five substrates, they were used at their saturating concentrations, i.e. 5 mM for phenylpyruvate and 10 mM for the other substrates. The chemostat cultivation, preparation of cell extracts and enzyme assays were performed as previously described by [36, 37]. The protein concentrations in cell extracts were estimated by the Lowry method [48] where bovine serum albumin was used as a standard.

### Statistical analysis

The presented values are averages of biological triplicates with standard errors. The differences between the measured volatile compounds were determined by a one-way ANOVA, followed by Tukey’s HSD test (statistical level of significance was set at *P* ≤ 0.05). The analysis was performed using the STATISTICA 7.0 software (StatSoft, Inc., Tulsa, OK, USA).

## Additional file

10.1186/s12934-016-0449-z List of *S. cerevisisae* and *S. kudriavzevii* strains involved in the bioinformatic analysis. The strains were obtained from corresponding databases as indicated.

## Abbreviations

*ADH1*-*7*: alcohol dehydrogenase 1–7 genes
*ARO10*: phenylpyruvate decarboxylase gene
*ATF1*: alcohol acetyltransferase 1 gene
*ATF2*: alcohol acetyltransferase 2 gene
*PDC1*: pyruvate decarboxylase isozyme 1 gene
*PDC5*: pyruvate decarboxylase isozyme 5 gene
*PDC6*: pyruvate decarboxylase isozyme 6 gene
Aro10p: phenylpyruvate decarboxylase
Atf1p: alcohol o-acetyltransferase
Atf2p: alcohol o-acetyltransferase
Iah1p: isoamyl acetate-hydrolyzing esterase
YNB: yeast nitrogen bases
