# Supplementary material for: Characterisation of the broad substrate specificity 2-keto acid decarboxylase Aro10p of Saccharomyces kudriavzevii and its implication in aroma development
Source: Microb Cell Fact. 2016 Mar 12;15:51. doi: 10.1186/s12934-016-0449-z (PMC4789280; doi:10.1186/s12934-016-0449-z)
Supplement: Supplementary file 1 — 10.1186/s12934-016-0449-z List of S. cerevisisae and S. kudriavzevii strains involved in the bioinformatic analysis. The strains were obtained from corresponding databases as indicated. [file 12934_2016_449_MOESM1_ESM.docx]

Additional file 1 List of *S. cerevisisae* and *S. kudriavzevii* strains involved in the bioinformatic analysis. The strains were obtained from corresponding databases as indicated.

| *S. cerevisiae* strains | | *S. kudriavzevii* strains |
| --- | --- | --- |
| SGRP database  ([www.moseslab.csb.utoronto.ca/sgrp](http://www.moseslab.csb.utoronto.ca/sgrp)) | SGD  ([www.yeastgenome.org](http://www.yeastgenome.org)) | *Saccharomyces* *sensu stricto* database  ([www.saccharomycessensustricto.org](http://www.saccharomycessensustricto.org)) |
| 273614N | AWRI1631 | IFO1802 |
| 322134S | AWRI796 | ZP591 |
| 378604X | BY4741 |  |
| BC187 | BY4742 |  |
| DBVPG1106 | CBS7960 |  |
| DBVPG1373 | CEN.PK |  |
| DBVPG1788 | CLIB215 |  |
| DBVPG1853 | CLIB324 |  |
| DBVPG6040 | CLIB382 |  |
| DBVPG6044 | D273-10B |  |
| DBVPG6765 | EC1118 |  |
| K11 | EC9-8 |  |
| L_1374 | FL100 |  |
| L_1528 | FY1679 |  |
| NCYC110 | FostersB |  |
| NCYC361 | FostersO |  |
| RM11_1A | JAY291 |  |
| S288c | JK9-3d |  |
| SK1 | K11 |  |
| UWOPS03_461.4 | Kyokai7 |  |
| UWOPS05_217.3 | LalvinQA23 |  |
| UWOPS05_227.2 | M22 |  |
| UWOPS83_787.3 | PW5 |  |
| UWOPS87_2421 | SEY6210 |  |
| W303 | Sigma1278b |  |
| Y9 | T7 |  |
| Y10 | T73 |  |
| Y12 | UC5 |  |
| Y55 | VL3 |  |
| YIIc17_E5 | Vin13 |  |
| YJM789 | X2180-1A |  |
| YJM975 | YJM269 |  |
| YJM978 | YJM339 |  |
| YJM981 | YPH499 |  |
| YPS128 | YPS163 |  |
| YPS606 | ZTW1 |  |
| YS2 |  |  |
| YS4 |  |  |
| YS9 |  |  |

SGRP – reference [41, 42], SGD – reference [40], *Saccharomyces sensu stricto* database – reference [29]
